# A Nosocomial Outbreak of Clinical Sepsis in a Neonatal Care Unit (NCU) in Port-Au-Prince Haiti, July 2014 – September 2015.

**DOI:** 10.1371/currents.outbreaks.58723332ec0de952adefd9a9b6905932

**Published:** 2018-03-21

**Authors:** Annick Lenglet, Olumide Faniyan, Joost Hopman

**Affiliations:** Médecins Sans Frontières, Operational Center Amsterdam, Public Health Department, Amsterdam, The Netherlands; Médecins Sans Frontières-Operational Centre Amsterdam, Port au Prince, Haiti; Médecins Sans Frontières, Operational Centre Amsterdam (OCA), Amsterdam, The Netherlands and Radboud University Hospital, Nijmegen, The Netherlands

**Keywords:** antibiotic resistant, disease outbreak, ESBL, Haiti, Klebsiella pneumoniae, nosocomial transmission

## Abstract

**Introduction:**

Between July 2014 and September 2015, a neonatal care unit (NCU) in Port Au Prince, Haiti, experienced an outbreak of sepsis, most probably due to nosocomial transmission of Extended Beta Lactamase (ESBL) producing gram negative bacteria, included Klebsiella pneumoniae.

**Methods:**

We describe the epidemiological and microbiological activities performed as part of the outbreak investigation and the control measures implemented throughout this period.

**Results:**

During the study period 257 cases of sepsis were reported, of which 191 died. The case fatality decreased from 100% in July 2014 to 24% in September 2015 and could be attributed to an improvement in clinical management and strengthened infection prevention and control measures. Risk factors identified to be associated with having late onset sepsis (sepsis onset >48 hours after birth)(n=205/257, 79. included: all categories of birthweight lower than <2500g (p=<0.0001) and all categories of gestational age younger than 36 weeks (p=0.0002). Microbiological investigations confirmed that out of 32 isolates (N=55; 58%) that were positive for gram negative bacteria, 27 (89%) were due to K. pneumoniae and most of these were from single MLST type (ST37).

**Discussion:**

This outbreak highlighted the importance of epidemiological and microbiological surveillance during an outbreak of sepsis in a NCU in a low resource setting, including regular point prevalence surveys.

## Introduction

The presence and spread of Extended–spectrum beta-lactamase (ESBL) producing Enterobacteriaciae is presently an issue of global public health concern. Not only are these bacteria resistant to several third generation cephalosporins, but ESBL-positive isolates display resistance to other antibiotics such as fluoroquinolones, aminoglycosides and trimethoprim-sulfamethoxazole which reduces the number of effective antibiotics available for treatment 1.

High rates of colonisation of with ESBL positive strains of *Klebsiella pneumoniae* have been observed in pediatric care units where 39% of isolated strains were ESBL positive 1 . High ESBL-positivity rates have also been identified amongst bloodstream isolates of* K. pneumoniae* from neonates in India (60%) and Algeria (80%) 2^,^^3^. A recent systematic review of ESBL producing Enterobacteriaciae in neonatal intensive care units (NICUs) showed that ESBL *K. pneumoniae* is the most frequently implicated pathogen in outbreaks in these contexts with mortality rates up to 31% in infected infants 4. The risk of high rates of colonization and possible infection and treatment options for treatment for ESBL positive Enterbactericiae has become established as a major challenge in the care of neonates and children in hospital settings in both high and low resource contexts.

Médecins Sans Frontières (MSF) runs an emergency obstetric hospital and associated neonatal care unit (NCU) in Port-Au-Prince, a low-resource setting. In July 2014, hospital pediatricians observed an increase in sepsis cases with a characteristic and not previously witnessed clinical presentation. Babies were initially observed to present with prolonged capillary refill (≥ 3 seconds), and a change of skin color. The babies appeared pale, often with a characteristic greenish tint. Other clinical signs observed included abdominal distension, a rise in temperature, and elevations in the heart rate and respiratory rate. Some affected neonates also developed signs of meningitis. The outbreak was characterized by a high mortality, particularly at its onset – all cases detected in the first three months died. Initial microbiological testing of blood cultures from septic infants resulted in isolation of ESBL positive *K. pneumoniae* from blood cultures.

We present our experience in investigating and managing an ongoing outbreak of sepsis. We have focused on the period between the onset of the outbreak (July 2014) and September 2015. We describe the efforts taken by the hospital in response to the outbreak and the analysis of information results from the epidemiological and microbiological surveillance activities.

## Methods

***Setting***

CRUO is a container hospital (established soon after the earthquake in 2010) and is equipped with 162 beds, including 106 obstetrics beds and 56 for neonates. The hospital runs on city power when available and is otherwise fully supplied by external generators. Water supply is done by a private company who provide trucked water supply. All water is filtered and chlorinated on site of the hospital to render it potable. Neonatal care relies on phototherapy, incubators and Continuous Positive Airway Pressure (CPAP) machines for neonates with breathing difficulties. Prophylactic antibiotic therapy is administered to all admitted neonates considered to be at risk for sepsis, based on prematurity, low birth weight, chorioamnionitis, or other materno-fetal risk factors. Neonatal care does not rely on central or umbilical catheters and thus we refer to the unit as an NCU.

Between July 2014 and September 2015, 7683 deliveries (mean=17 daily) occurred at CRUO with 3380 neonatal admissions to the NCU (mean admission time = 8 days). Half (53%) of admitted infants had a low birthweight (LBW) (< 2.5 kg) and 35% were premature (gestational age at birth < 37 weeks). Routine medical data collection until July 2014 did not give us the ability to determine historical incidence of sepsis, thus no background rates were available at the time of the outbreak intervention.

***Case definition***

A suspected case of sepsis for retrospective chart review was defined as a neonate in the NICU who experienced prolonged capillary refill (≥3 seconds), a distended abdomen and tachypnea. A suspected case of sepsis for prospective surveillance (and thus based on clinical diagnosis) was defined as a neonate in the neonatal unit who presented with one or more of the following clinical signs: prolonged capillary refill, certain skin changes (redness, sclerema), distended abdomen with/without hemorrhagic, brownish or bilious gastric aspirates, tachypnea, tachycardia, persistent jaundice, unstable temperature, signs of disseminated intravascular coagulation (bleeding from catheter sites, bloody secretions from nose and mouth, petechiae), reduced muscle tone, lethargy, apathy or irritability. In the clinical case definition there we did not distinguish between early onset (EOS) and late onset sepsis (LOS). However, in the analysis of epidemiological data we assumed a clinical case of sepsis that occurred less than two days after admission to the NCU was a case of EOS.

A confirmed case of *K. pneumoniae* infection was an infant with suspected sepsis for whom *K. pneumoniae* was isolated from their blood culture. Other bacterial isolates from blood culture were also classified based on species, ESBL status and antibiotic resistance profiles.

***Microbiological investigations***

Standardized microbiological testing criteria were introduced in early October 2014. Blood cultures were taken whenever possible for infants who were considered suspected cases of sepsis. Blood cultures were plated on MacConkey, CAN and PVX plates and incubated for 24 hours at 37°C. The isolates were identified using the Vitek2 Gram-negative (GN) card, and antimicrobial susceptibility testing was performed using the Vitek 2 AST-N233 card (Biomérieux, Durham, NC, USA). Presence of ESBL as well as other forms of resistance was established through a synergy test using Mueller Hinton Agar with impregnated antibiotic discs for Beta-lactamase detection or confirmation. Amoxcillin/clavulanic was placed in the middle of the Agar, surrounded by discs for third and fourth generation cephalosporins (ceftazidime, cefotaxime, ceftriaxone and cefepime).

Multilocus sequence typing (MLST) was performed for a subset of *K. pneumoniae* isolates. DNA extracts were prepared by mixing 50 μl of the frozen isolate with 500 μl of nuclease-free water. A polymerase chain reaction (PCR) was performed using primers and conditions described by Diancourt et al.^5^. PCR fragments were analysed on 2% agarose gel and purified using the QIAquick PCR purification kit (QIAGEN, Hilden, Germany), pre-mixed with universal sequencing primers listed on the Klebsiella MLST website 6 [6] and sent for Sanger sequencing (3730XL) to Cornell University Bio Resource Center, Ithaca, NY, USA. Allele and type numbers were determined from sequencing reads on the Klebsiella MLST website.

***Response to the outbreak***

The outbreak response included strengthening infection prevention and control (IPC), improving clinical management, and revising antibiotic treatment algorithms, establishing standard operating procedures (SOPs) for sampling of septic infants and maintaining epidemiological and microbiological surveillance. In February 2015, a “septicemia committee” was established to ensure a more coordinated approach to the multi-sectoral outbreak response. Meetings of this committee were held (and have since continued) at least fortnightly.

A series of IPC measures were gradually introduced during the reported outbreak. Firstly, hand hygiene protocols were strengthened to encourage appropriate disinfection of hands by staff and parents/caretakers visiting neonates. Alcohol gel for hand hygiene was available in the NCU at the time of the start of the outbreak, but supplies were increased so as to ensure a single bottle of alcohol gel on each neonatal cot, as well as at the entrance of each ward in the paediatric department. Transmission based precautions which were strengthened or implemented and included: obligatory donning of gowns upon entrance in the pediatric wards (and removal upon leaving) and introduction of single use medical equipment for CPAP, suction bulbs and nasal prongs. Additionally, disinfection and cleaning protocols for a number of materials were improved. This included regular disinfection of frequently touched surfaces (including the external surfaces of all incubators) and deep cleaning of all incubators between babies. Infrastructure improvements were adopted to improve patient care, patient flow and improve space between neonatal cots. This included changing the incubator models in use so as to have incubators that did not contain water, allowing only one neonate on the pediatric admission table at any time, single use incubators, construction of new buildings to increase space in the pediatric wards and the replacement of all wooden furniture by furniture with cleanable surfaces.

In terms of clinical management, neonates recognised with suspected sepsis were immediately isolated in a separate isolation room. The protocols used for the preparation of intravenous fluids (IV) were adapted so as to have a single healthcare staff responsible for the preparation of all required IV fluids during a single shift. Antibiotic treatment regimens were also adapted. First line antibiotics in neonatal care remained as ampicillin and gentamicin, but second line antibiotics were changed from cloxacillin and cefotaxime to amikacin in October 2014, then to amikacin plus ceftazidime in November 2014. No third line treatment for sepsis was available until April 2015 when meropenem was introduced for this purpose. Many admitted neonates were already on first line antibiotic prophylaxis due to the risk factors they had upon admission. As soon as these neonates developed any clinical signs indicative of suspected sepsis, antibiotics were switched to second line, and subsequently to third line when deemed necessary.

Human resource and budget plans were adjusted to account for staffing and infrastructure changes. New nursing staff supervisors were hired to ensure permanent presence of senior medical staff to reinforce hygiene, infection control and clinical management procedures. Initial plans were drawn up to expand the available floor space for the NCU, so as to improve the spacing of beds, improve patient flow and increase the number of cleanable surfaces being used.

A target was set to minimise the moments where the neonatal bed occupancy exceeded 80%. Thus, collaborations were established with other hospitals in the city to allow for faster discharge of neonates that required longer term Kangaroo Mother Care (KMC), in order to liberate beds in the neonatal department.

****Surveillance****

An epidemiological linelist was initiated from July 2014. Between July and October 2014, this linelist only contained data on deaths from suspected sepsis. From November 2014, the linelist included all suspected cases and collected information on the gestational age, birth weight, Apgar scores, delivery mode of the babies, history of antimicrobial use, in addition to information regarding the use of continuous positive airway pressure (CPAP) and the administration of resuscitative procedures at birth. The linelist also contained the results of microbiological and susceptibility testing. The date when the baby was switched to second-line antibiotics was included as a proxy for the date of onset of sepsis. This linelist was used in addition to the routine medical monitoring tools which collect demographic and clinical information on all admitted neonates in the hospital.

***Data analysis***

Data from the sepsis line-list was merged with routinely collected medical data and was analysed with Stata 12.1 (College Station, TX, USA). We compared characteristics (gestational age, birth weight, length of stay, Apgar scores at birth) of suspected LOS cases to all other neonatal admissions in the NCU during the same study period. We compared odds of exposure between cases and non-cases using unadjusted logistic regression to calculate Odds Ratios (ORs) and respective 95% confidence intervals (95%CI). We calculated adjusted ORs for those factors that were associated with sepsis in the unadjusted regression in a multivariate logistic regression model.

We calculated means, medians, ranges and frequencies to describe characteristics of suspected sepsis cases and confirmed cases K. pneumoniae. For bacterial isolates we calculated the proportion of isolates from blood samples for different antibiotic resistance profiles and ESBL status.

## Results

Between July 2014 and September 2015, we identified 257 cases of suspected sepsis cases. The incidence of clinical sepsis decreased from 50 sepsis cases per 1000 patient-days in July 2014 14.6 sepsis cases per 1000 patient days in September 2015 (Figure 1). Most of the sepsis cases occurred during two high peaks of sepsis in 2015 during July and October (n=174, 68%) (Figure 1). In total, 191 cases died. The case fatality decreased substantially throughout the study period from 100% in July 2014 (based also on the fact that only sepsis deaths were recorded during this period) to 21% between July and September 2015 (Figure 2). Of the 257 cases of sepsis, 53 (20.6%) were classified as EOS and 204 (79.4%) as LOS.

**Epidemic curve of sepsis cases, confirmed cases of K. pneumoniae, gram negative bacteria cases and incidence of sepsis between July 2014 and September 2015.**
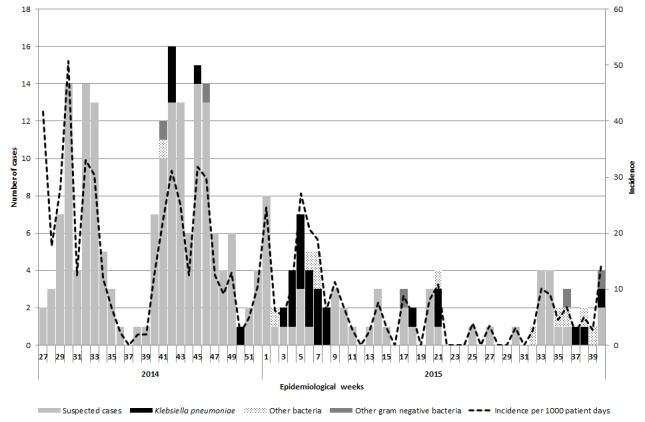

**Case fatality ratio of suspected cases of clinical sepsis between July 2014 and September 2015.**
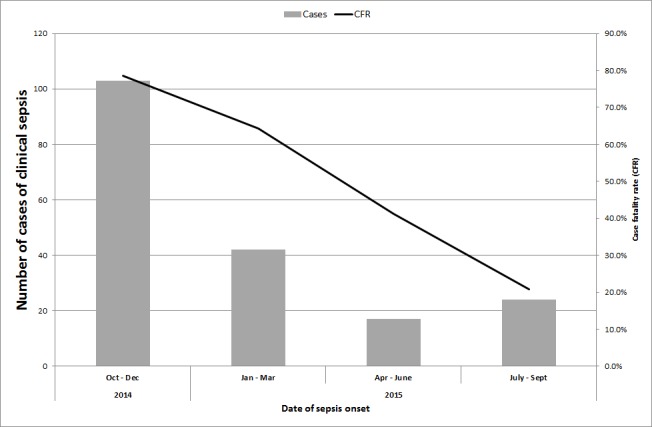
NB: CFR from July to September 2014 is not represented as all data recorded during this time period included only deaths.

***Risk factors for LOS***

The sex distribution between LOS cases and all other admissions in CRUO (excluding the EOS cases) during the study period were similar (Table 1). Out of all LOS cases (n=204), 76.0% (n=155) were younger than 36 weeks of gestational age, compared to 65% of non-sepsis admissions being 37 weeks of gestational age or older. All categories of gestational age younger than 36 weeks were associated with being an LOS case (p<0.001). Half of non-sepsis cases (n=1563) were considered low birth weight (<2500g) compared to 87.7% (n=179) of LOS cases. All categories of LBW were independently associated with being an LOS case (p=0.001). Having APGAR scores below 8 at five and 10 minutes following birth were associated with being an LOS case in the unadjusted analysis (p<0.0001), but there was less evidence for an association in the adjusted analysis (OR=1.5; CI95%=1.0-2.3 and OR=0.9; CI95%: 0.5-1.4 respectively)(Table 1). Similarly, receiving cardiac or respiratory resuscitation at birth was not associated with being an LOS case in the adjusted analysis (OR=1.5, CI95%=0.7-3.1). Receiving CPAP at birth was not associated with being an LOS case (OR=1.2, CI95%: 0.2-9.2). There was some evidence to suggest that being delivered by Caesarean section increased the risk of being an LOS case in the unadjusted and adjusted analysis but these ORs were not statistically significant (Table 1).

**Table 1: Unadjusted and adjusted analysis for risk factors associated with LOS, CRUO, July 2014- September 2015.** * Included in the multivariate logistic regression model; ^a^ Defined as as an Apgar score of less than 7.

		Non sepsis cases (N=3117)		LOS cases (N=204)		Unadjusted analysis			Adjusted analysis		
		n	%	n	%	OR	CI95%	p-value	aOR	CI95%	p-value
Sex	Male	1,637	52.5	98	48	1.2	0.9-1.6	0.2	0.8	0.6-1.2	0.2
	Female	1,458	46.8	105	51.5						
	Missing	22									
Gestational Age *	≥37 Weeks	2,016	64.7	49	24.0	Ref		<0.0001	Ref		<0.0001
	34-36 Weeks	415	13.3	36	17.7	3.6	2.3-5.6		2.1	1.1-3.8	
	29-33 Weeks	410	13.2	96	47.1	9.6	6.7-13.8		4.0	2.2-7.2	
	≤28 Weeks	147	4.7	23	11.3	6.4	3.8-10.9		1.3	0.5-3.4	
	Missing	129	4.1	0	0						
Birth weight*	≥2500g	1,549	49.7	25	12.3	Ref		<0.0001	Ref		0.001
	2000-2499g	526	16.9	27	13.2	3.2	1.8-5.5		2.1	1.1-4.1	
	1500-1999g	515	16.5	48.	23.5	5.8	3.5-9.4		3.0	1.5-6.1	
	1000-1499g	360	11.6	77	37.8	13.3	8.3-21.1		4.8	2.3-9.7	
	<1000g	162	5.2	27	13.2	10.3	5.9-18.2		3.7	1.5-9.3	
	Missing	5	0.2	0	0						
Low Apgar 5 minutes*a	No	1,975	63.4	91	44.6	2.2	1.7-3.0	<0.0001	1.5	1.0-2.3	0.07
	Yes	989	31.7	101	49.5						
	Missing	153	4.9	12	5.9						
Low Apgar 10 Minutes*a	No	2,016	64.7	115	56.4	1.8	1.2-2.6	0.002	0.9	0.5-1.4	0.6
	Yes	413	13.3	42	20.6						
	Missing	688	22.1	47	23.0						
Resucitation at birth*	No	3,017	96.8	189	92.7	2.0	1.1-3.6	0.03	1.5	0.7-3.1	0.3
	Yes	98	3.1	12	5.9						
	Missing	2	0.1	3	1.5						
CPAP	No	2,981	95.6	191	93.6	1.2	0.2-9.2	0.9			
	Yes	13	0.4	1	0.5						
	Missing	123	4.0	12	5.9						
Route delivery*	Vaginal	1,200	38.5	78	38.2	1.3	1.0-1.8	0.1	1.3	0.9-1.9	0.1
	Caesarian Section	1,405	45.1	122	59.8						
	Missing	512	16.4	4	2.0						

****Microbiological findings****

A total of 96 biological samples were taken during the study period from 49 suspected sepsis cases (19%). Of these, 55 (57.3%) were blood samples, 40 (41.7%) were rectal or peri-anal swabs and one was an ear swab. We have focused the analysis on the results from the blood sample isolates.

In the 55 blood samples taken (corresponding to 49 neonates), 32 were positive for gram negative bacteria (58%), six were positive for other pathogens (11%), 16 were sterile (29%) and one sample did not have a result available. For the gram negative bacterial isolates, 27 (49% of all isolates and 84% of all gram negative bacteria isolates) were positive for *K. pneumoniae*, corresponding to 27 individual sepsis cases (10.5% of all suspected cases and 55% of all sepsis cases for whom a blood isolate was available). Twenty-two out of 27 isolates (81.5%) of *K. pneumoniae* were isolated from blood cultures of LOS patients. The confirmed cases were identified at all stages of the outbreak (Figure 1). The other gram-negative isolates (n=5, corresponding to five individual neonates) were due to *Enterobacter aerogenes*, *E. cloacae*, *Pseudomonas aeruginosa*, *Serratia marcescens* and *Serratia odorifera*. For the other bacterial isolates, one was due to *Staphylococcus haemolyticus*, one was labelled as ‘negative for *K. pneumoniae*, and four were from suspected contamination with *Staphylococcus epidermis* and *Staphylococcus hominis*.

****ESBL prevalence and antibiograms for gram negative bacterial isolates****

Out of the 32 gram-negative bacterial isolates from blood culture, 24 (75%) had data available on ESBL status and they were all ESBL positive (100%); 22 were *K. pneumoniae* isolates. Out of the 26 isolates tested for gentamycin (first line treatment) resistance, two remained sensitive to this antibiotic (7.7%). Out of the 25 isolates tested for third generation cephalosporins (i.e. cefotaxime even though ceftazidime is used as second line treatment for sepsis in CRUO), hree (12.0%) were sensitive. All of the isolates were sensitive for amikacin (second line treatment) (n=27, 100%) and imipenem (third line treatment is meropenem) (n=26, 100%).

Multi-locus sequence testing (MLST) was carried out on 15 bacterial isolates of *K. pneumoniae* isolated from septic neonates with dates of onset between January and February 2015. Nine were identified as ST37, one was identified as ST39 and the remainder as other types. A second round of testing was carried out on isolates of 13 septic babies with dates of onset between March and April 2015. Four were identified with ST39 and the remainder consisted of either differing ST-types or non-typeable samples.

***Ethical considerations***

This research fulfilled the exemption criteria set by the MSF ERB for a posteriori analyses of routinely collected clinical data and thus did not require MSF ERB review (http://fieldresearch.msf.org/msf/handle/10144/618714). It was conducted with permission from the Medical Director.

## Discussion

The occurrence of sepsis in neonates has been recognized as a common morbidity in Haiti before 7. However, this is the first time that an outbreak of sepsis due to ESBL positive gram negative bacteria, specifically *K. pneumoniae* is reported from this NCU in Haiti. Similar outbreaks have also been reported in the region 8^,^^9^ and neonatal intensive care units in other lower resources settings 4^,^^10^^,^^11^^,^^12^.

During the study period the outbreak demonstrated two peaks of high incidence and then sporadic smaller peaks of activity. The risk factors identified for the neonates acquiring LOS included low gestational age and low birth weight. The inverse relationship between late-onset neonatal sepsis and gestational age and birth weight has been previously demonstrated 13. Unfortunately, none of the risk factor analysis led to any crucial indications around possible transmission routes or sources of transmission. This analysis only confirmed that the youngest, smallest and most vulnerable neonates admitted at the hospital were most at risk of getting LOS.

We were unable to identify a single source of transmission and thus assumed that the majority of infections with gram negative bacteria were due to weak infection and hygiene measures. Most likely, transmission was due to contaminated hands from staff and parents/caretakers and infection was facilitated in vulnerable small neonates. As the measures implemented to address contamination of surfaces and hands were multi-fold, the reduction in the high incidence peaks of sepsis observed in the hospital, cannot be attributed to a single measure that was implemented during the study period.

However, part of it is likely explained by a re-enforced infection control strategy throughout the NCU including the use of alcohol based gel for hand hygiene, isolation, improved spacing between beds and improved staffing numbers (and trained staff). The results demonstrate that a combination of these multi-modal measures is effective in improving outcomes with neonatal sepsis.

The high initial case fatality observed in septic infants in our unit can be attributed to the limitations around data collection at the early part of the outbreak and that only deaths were being recorded. Even so, the decline in mortality of septic neonates was slow and steady throughout the outbreak. The overall case fatality in septic neonates during this outbreak was 74% which is higher than the mortality reported in a recent meta-analysis of ESBL sepsis outcomes, which included cases from lower resource settings 4.

However, high mortality rates (64%)were also reported from similar outbreaks in Guatemala 9. We attribute the progressive decline in mortality in septic neonates to improved surveillance data, an increased awareness in healthcare staff to identify the unspecific initial symptoms of sepsis early and therefore start prompt treatment. The improved clinical management can also be attributed to the availability of second line treatment of amikacin to which the gram negative bacteria were still resistant and our ability to acquire meropenem as third-line treatment.

Compared to most low resource settings, we had the luxury in Haiti to have access to high quality microbiological testing for blood and fecal samples. This allowed us to encourage systematic microbiological surveillance for bacterial isolation, antibiograms and ESBL identification. Additionally for a sub sample of isolates early in the outbreak we were able to show that an ESBL-positive *K. pneumoniae* of a single MLST type was most likely responsible for several of the high peaks of sepsis observed in the early part of the outbreak. The majority of sepsis cases were LOS. This together with the isolation of several other gram negative bacteria (from blood cultures of LOS cases) during the same period strongly suggests that nosocomial transmission with gram-negative bacteria of all types was regularly occurring in this NCU, possibly from different sources of contamination or at different times. It is very likely that similar pathogens, with similar resistance profiles are present in other health facilities managing neonates in other lower resources settings (including Haiti), thus serves as evidence that infection control and antibiotic stewardship are crucial aspects of hospital management in Haiti.

We must however note a series of limitations which have reduced our ability to investigate the outbreak more comprehensively and identify a possible source of transmission. Systematic data collection around indicators such as mortality, treatment outcomes, patient exposures to risk factors for sepsis (i.e. previous antibiotic use, staffing levels, and patient bed locations within the NCU etc.) and microbiological testing of environmental samples fell short during this outbreak to help identify transmission routes. The surveillance bias for deaths at the start of the outbreak caused a likely overestimation of the CFR. The low success rate in taking blood cultures from septic infants, despite access to a high quality laboratory, limited our ability to track the dynamics of the outbreak in microbiological terms. We must emphasize that this was the first time the hospital was confronted with a situation in which microbiological analysis of blood cultures of the neonatal admissions was systematically required. After the study period described, even additional effort was invested in improving the proportion of septic infants for who blood cultures were taken. Furthermore, the absence of other data has fed directly into the review of our Health Information Systems for pediatric departments and a more comprehensive number of variables are currently available in this hospital for future outbreak response.

The World Health Organisation recently published a list of twelve priority pathogens for which new antibiotics are needed urgently^14^ . This list included the ESBL positive gram negative bacteria that were identified in the currently described outbreak in Haiti. Thus, this outbreak description is a stark reminder of that urgency and the risk that these bacteria are also representing in lower-resource healthcare facilities. Epidemiological and microbiological surveillance in low resource settings such as this hospital are crucial to improving clinical management and adapting treatment regimens, implementing immediate infection control measures, and improving our understanding and ability to prevent and manage such infections in the future. Further research on this topic should focus on further identifying the effectiveness of individual specific infection control measures in curtailing outbreaks and improving outcomes of infected patients.

## Funding

All investigations were funded by MSF-OCA as these were part of routine activities in relation to the functioning of the hospital. The funders had no role in study design, data collection and analysis, decision to publish, or preparation of the manuscript.

## Competing Interests

The authors have declared that no competing interests exist.

## Corresponding Author

Annick Lenglet: annick.lenglet@amsterdam.msf.org

## Data availability statement

Médecins Sans Frontières (MSF) has a managed system for data sharing. Data are available on request in accordance with MSF's data sharing policy. Requets for access to data should be made to data.sharing@msf.org.
